# The Peacock Fallacy: Art as a Veblenian Signal

**DOI:** 10.3389/fpsyg.2021.767409

**Published:** 2021-11-22

**Authors:** Larissa Mendoza Straffon

**Affiliations:** ^1^Department of Psychosocial Science, University of Bergen, Bergen, Norway; ^2^Centre for Early Sapiens Behaviour, University of Bergen, Bergen, Norway; ^3^Cognitive Psychology Unit, Leiden University, Leiden, Netherlands

**Keywords:** art, sexual selection, mate choice, costly signaling, the handicap principle, visual art, Veblen

## Abstract

The fact that world-over people seem inexplicably motivated to allocate time and effort to apparently useless cultural practices, like the arts, has led several evolutionary scholars to suggest that these might be costly Zahavian signals correlated with genetic fitness, such as the infamous peacock’s tail. In this paper, I review the fundamental arguments of the hypothesis that art evolved and serves as a costly Zahavian signal. First, I look into the hypothesis that humans exert mate choice for indirect benefits and argue that the data supports mate choice for direct benefits instead. Second, I argue that art practice may well be a costly signal, however not necessarily related to good genes. Third, I suggest that Thorstein Veblen’s original concept of conspicuous signals as social tools to obtain and convey prestige provides a better account than the Zahavian model for the evolution and function of art in society. As a Veblenian signal, art could still have many of the effects suggested for visual art as a Zahavian signal, except not for the indirect benefits of optimal offspring, but for the direct benefits of acquiring and conveying social status.

## Introduction

For years, the tail of the peacock posed a problem for Darwin’s principle of natural selection, so much so that in 1860 he wrote to a friend that its mere sight made him sick (Hiraiwa-Hasegawa, 2000). Conspicuous animal traits like the peacock’s tail or the deer’s antlers had eluded Darwin because they did not seem to contribute towards the survival of the individuals that possessed them. On the contrary, sometimes such traits seemed to hinder survivorship, raising the question of why these evolved at all. Darwin eventually arrived at the mechanism of sexual selection to explain those exaggerated characters either as armaments or ornaments employed in rivalry battle and courtship displays, respectively (Andersson, 1994).

Sexual selection has provided a successful theoretical framework in evolutionary biology. It has offered an explanation for many animal traits and behaviors, from birdsong to tusks and horns.^1^ These characters do not usually aid survival but are essential in mating displays and rival confrontation. In addition, their presence and salience tend to correlate with the animal’s overall health, thus they are hypothesized to serve as indicators of individual fitness. The classic example is the peacock’s tail, whose large, colorful, eye-spotted feathers incur a huge energetic investment. While attractive to the peahens, the tail makes the male bird less agile and more noticeable to predators. So, those peacocks that despite the handicap of the costs and risks of the tail are still able to sustain and display it conspicuously should be perceived as high-quality mates and preferred by the peahens, whose offspring will inherit both the elaborate tail (the males) and a preference for it (the females; Zahavi, 1975; Jones and Ratterman, 2009).^2^ This is broadly the basis of the so-called “Handicap Principle” (Zahavi and Zahavi, 1997), also known as “fitness indicator” model of sexual selection.^3^

People across all periods and cultures have inexplicably allocated much time and effort to cultural practices which seem useless to survival, like the arts. This has led several evolutionary scholars to suggest that art, too, might have evolved through sexual selection (Taylor, 1996; Zahavi and Zahavi, 1997; Kohn and Mithen, 1999; Miller, 2000; Thornhill, 2003; Dutton, 2009). The arts, and particularly visual art, are often referred to as costly “Zahavian” signals associated to genetic fitness, or “good-gene indicators” (Miller, 1999). From this perspective, even if art did not increase a person’s survival chances, it would increase their mating opportunities and, like the peacock’s tail, would serve to outcompete rivals and impress the opposite sex. Therefore, just like those exuberant animal traits, art might be considered a courtship adaptation (Miller, 2000).

This argument has had a huge impact, particularly in popular science. It has been frequently reproduced and applied to various human cultural behaviors, from music (Bolt, 2008) to literature (Gottschall et al., 2004; Gottschall, 2008), to visual art (Dutton, 2009). Similarly, it has been criticized for being too broad and oversimplistic, and for lacking empirical support (Brown, 2000; Carroll, 2004; Fitch, 2005; Dissanayake, 2007; Boyd, 2009; Straffon, 2019). Such critiques, however, have often been limited to the implications of the model for art studies, but have not dealt with the underlying arguments. In this paper, I review and assess the fundamental premises of the hypothesis that visual art evolved as a human fitness indicator, or Zahavian signal. I focus on three key arguments. The first is that humans make use of good-gene indicators to guide their mating choices. Second, that art is a biological signal of genetic fitness. Third, that because art has no survival function, it makes no evolutionary sense except as a sexually selected trait. I further discuss that although the hypothesis is appealing on the surface, it offers no sound argument for either the evolution of art or its role in human mating behavior. Alternatively, I suggest that art may indeed serve as a costly signal, but rather in Veblen’s original sociocultural sense.

## Art as a Costly Zahavian Signal

Sexual selection can occur through different mechanisms (see Table 1), including the two discussed by Darwin, rival contests and mate preference. The hypothesis of art as a Zahavian signal focuses on the latter, mate choice, which refers to “the outcome of the inherent propensity of an individual to mate more readily with certain phenotypes of the opposite sex (i.e., mating preference or bias) and the extent to which an individual engages in mate sampling before deciding to mate (i.e., choosiness)” (Kokko et al., 2006, p. 49). Mate choice is of special interest precisely because it seems to be directly correlated with the evolution of the ornaments and the extravagant traits which Darwin struggled to explain through natural selection (Kokko et al., 2003).

**Table 1:** The different mechanisms of sexual selection and the traits they favor in the competing sex.

Mechanism	Traits favored in the competing sex
Scrambles	Early search and swift location of mates; well-developed sensory and locomotor organs
Endurance rivalry	1. Traits that improve fights (e.g., large size, strength, weaponry, agility or threat signals) 2. Alternative mating tactics of inferior competitors, avoiding contests with superior rivals
Mate choice	1. Behavioral and morphological traits that attract and stimulate mates 2. Offering nutrition, territories, nest sites or other resources needed by the mate for breeding 3. Alternative mating tactics, such as forced copulation
Coercion	1. Similar traits as for contests (1) 2. Morphological and other adaptations for forced copulation and other coercive behavior
Sperm competition	1. Mate guarding, sequestering, frequent copulation, production of mating plugs or other means of preventing rivals from copulating with mate 2. Ability of displacing rival sperm; production of abundant sperm to outcompete those of rivals
Infanticide	Similar as traits for contests (1)

After Andersson and Iwasa (1996).

Parental investment theory proposes that there is some conflict of interest between males and females since they invest unevenly in the offspring, leading to different mate choice strategies (Trivers, 1972). Because usually the females invest more in reproduction and parental care, they will tend to be the choosier sex, whereas the males will compete with each other for mating opportunities. Therefore, the most common mating dynamics involve male–male competition and female choice (Geary et al., 2004), although these two by no means exhaust the wide array of mate choice behaviors and mechanisms (Andersson and Simmons, 2006; Jones and Ratterman, 2009; Rosenthal, 2017).

Here I will only discuss the two forms that are most relevant for understanding Zahavian signals: mate choice for direct benefits and mate choice for indirect benefits. In the first, individuals select mates for an *immediate fitness advantage*, that is, for direct phenotypic effects such as the procurement of resources, territory, parental care, protection, fertility, health, etc. In the second case, individuals choose mates for *indirect benefits* (fitness advantages passed down to the offspring) based on an indicator trait that correlates with the desired advantage, for example an ornament like bright plumage. In addition to selection strategies for benefits, mate choice may be based on some perceptual preference originated in a non-sexual context (sensory bias; Andersson and Simmons, 2006; Jones and Ratterman, 2009),^4^ or on social information (mate copying; Dugatkin, 1992; Mery et al., 2009).

The hypothesis of art as a Zahavian signal relies on mate choice for indirect benefits, where the preferred trait is assumed to be a reliable indicator of the individual’s overall genetic quality, which would subsequently be inherited by the offspring. In the case of the peacock, the tail is correlated with the general physical condition of the male and should be more elaborate in strong, healthy individuals. The peacocks that despite the costs and risks can still afford to maintain and display their tail conspicuously will be preferred by the peahens as high-quality mates. The peacock’s tail is hence described as a wasteful or costly fitness signal (Zahavi, 1975). In the human case, several cultural behaviors such as humor, music, art, and altruism have been hypothesized to have evolved precisely like the peacock’s tail, as fitness indicators for a courtship function (Miller, 2000).

When discussing the effects of sexual selection in *The Descent of Man*, Darwin himself alluded to a probable correlation between the human “passion for ornament” and the affairs of choosing a mate. He suggested that, just as the vivid colors and patterns of some male birds serve them to lure females, humans turn to decoration to enhance their natural qualities and make themselves more attractive to the opposite sex (Darwin, 2004).

As stated above, several authors have followed up on Darwin’s observation, and suggested that human aesthetic production is costly and wasteful, requiring energy and resources that had better be invested in survival efforts like foraging, rest, or defense. The argument is that natural selection generally is an economizing process that does not promote the persistence of useless behavior, so it cannot explain art-making. Sexual selection, on the other hand, often results in the development of exaggerated and seemingly wasteful but attractive traits, like the plumage of the birds-of-paradise. A strategy of mate choice that selects for indicators of good genes therefore provides a reasonable framework to explain the evolution of visual art (Zahavi and Zahavi, 1997; Miller, 2000).

Human mating preferences and mating strategies have received much attention in evolutionary psychology. Unlike social scientists who typically claim that mating choices are a function of socio-economic pressures and culturally shaped gender roles (Wood and Eagly, 2002), evolutionary psychologists favor the view that mating preferences are innate psychological adaptations that guide individuals in choosing high-quality partners (Gangestad et al., 2006). Likewise, mating strategies are seen as the result of the reproductive problems faced by humans throughout evolution (Buss, 1994), such as whether to invest in offspring quantity or quality, or whether to invest in parental care or in multiple mates, etc. As in most mammals, human mating strategies are constrained by parental investment, thus women are expected to be choosier, and men are expected to engage in sexual rivalry and prowess displays (Buss, 1994).

According to the hypothesis under discussion, visual art would have evolved in the context of human mating strategies to serve a courtship function by signaling the artist’s fitness. The mental and physical abilities required for art-making (e.g., creativity, concentration, coordination, dexterity, etc.) would correlate with the general condition of the individual, serving as reliable indicators of good genes (Kohn and Mithen, 1999; Miller, 2000). The assumption is that traits which incur high energetic or intellectual costs function best as markers of genetic quality. So, a complex behavior like art-making which is hard to acquire, costly to maintain, and susceptible to the general physical and mental condition of the individual would function as a Zahavian signal, or good indicator of the creator’s fitness.

## Challenging Good-Genes Models

As in the example of the peacock, many of the assumptions of the good-genes model of art are based on the mating behaviors of phylogenetically distant species, typically birds, whose mate choice strategies are dominated by a pattern of male display and female choice, in which the males offer little or no parenting effort (Jones and Ratterman, 2009). These can shed light on the selective pressures that give rise to mating strategies across lineages. However, to understand and explain human mate choice it would be more parsimonious to look within the primate pattern. Unlike the peacock, primate males usually provide some parental care, and primate mating strategies vary from monogamy to polygamy and polyandry (Fuentes, 1999). Humans, for their part, fit within the pair-bonded primates, in which the male-female bond lasts beyond copulation and involves both a reproductive and a social partnership (Aureli et al., 2008). The evolutionary history of primate pair-bonding is closely related not to fitness indicators, but to mate choice for direct benefits – e.g., territoriality, protection, and sexual selection mechanisms other than mate choice such as intra-sexual competition (van Schaik and Dunbar, 1990).

The study of human mating systems in evolutionary psychology has traditionally assumed that good-gene traits like attractiveness and mental ability guide partner preference in humans, particularly in women (Buss, 1994; Miller, 2000). These traits can certainly play a part in mate choice (Gangestad et al., 2006), but sexual selection for good genes remains controversial even among non-human animals, with few empirical studies having provided support for fitness indicator models, moreover existing examples of fitness indicator traits could be interpreted in different ways (Andersson, 1994). Even the peacock’s tail may be seen as an evolved armament for intra-sexual competition rather than an ornament for courtship (Berglund et al., 1996), and may be uncorrelated to mating success (Takahashi et al., 2007). Many other traits that are supposed to serve as good-gene indicators might be equally correlated with some non-genetic direct phenotypic benefit to the female or the offspring (see Table 2).

**Table 2:** Frequently cited good-genes indicator traits and their alternative direct benefits interpretation.

Good-genes indicator trait	Direct benefit
Symmetry (both sexes)	Health, disease avoidance
Big size, strength (male)	Protection, territory
Low waist-to-hip ratio (female)	Immediate fertility
Intelligence (male)	Resource acquisition

The apparent preference for fitness indicators can in many cases be at least equally explained by mate choice for direct benefits. For example, anthropologists Hawkes and Bliege Bird (2002) have suggested that human hunting might have evolved primarily as a form of costly male display, and not for meat provisioning, a scenario also suggested by evolutionary psychologist Miller (1998). In their study, Hawkes and Bliege Bird clearly show that hunting is a central arena for male competition in forager societies. Good hunters have a high social status and often father more children than other men. On that basis, the authors advocate that hunting is a costly signal, or handicap, that works as a reliable indicator of male genetic quality. However, they fail to note that their work also reveals that whereas better hunters do seem to have more offspring, the survival rates of their children is not particularly higher, meaning that there is no actual fitness advantage for good hunters. In contrast, research shows that children of prestigious men with better access to resources in general do have a better chance of survival (Dunbar, 2012). Hunting may truly be a form of male contest but that need not support an indirect benefits model. Women could be choosing better hunters as mates not for their good genes, but for the direct benefits of high social status and their ability for securing provisions (Marlowe, 2005). The latter is compatible with data indicating that fertility and fitness are greatly influenced by resource allocation to women, because this will determine their available energy for reproduction and parental investment (Harris and Ross, 1987; Kaplan, 1996). It would then be more adaptive for women to select partners for direct benefits. Furthermore, mate choice for direct benefits matches observations that human reproduction involves investing not only in the quality of the offspring but also in reducing the risk of early mortality (Kaplan and Bock, 2001; Hopkinson et al., 2013).

Similar to the costly hunting example, a study originally designed to show that creativity was a desired trait in a potential mate (Clegg et al., 2011), in fact ended up showing that artistic success, measured as high social status, was what people valued as an attractive trait, not artistic creativity or skill, as predicted by a fitness indicator model. So, even if women preferred artistic types (Miller, 2000), it would probably be due to the status of artists in today’s society rather than to a supposed universal preference for creativity.

In a series of studies, Sundie et al. (2011) found that men more often displayed conspicuous consumption behaviors in contexts that provided potential short-term mating opportunities (i.e., copulation without parental investment), and inversely, women responded to such cues assessing those men as more attractive as short-term partners. But even if short-term mating was pursued through conspicuous consumption, this does not imply a match between showing off, male genetic fitness, and female mate preference. To make the case that human conspicuous consumption is equivalent to a biological handicap, traits related to this behavior, such as high testosterone levels and social dominance, would need to be related to heritable genetic fitness, and it would need to be demonstrated that such traits actually increase the survival and reproductive success of the offspring, none of which has been confirmed so far. Quite the opposite, high testosterone levels in birds have been associated with poor male parenting which indirectly decreases the fitness of the offspring (Reed et al., 2006). Likewise, children of dominant men who provide no parental care may be worse off, since paternal absence in general can have an adverse effect in child survivorship particularly in the early years (Hurtado and Hill, 1992). It is therefore unlikely that women would have evolved and sustained a preference for showing off as a good-genes indicator when it confers no genetic fitness advantage. On the other hand, even in the absence of male parental care, the resources provided by men are a strong predictor of children’s health and women’s fertility and well-being (Kaplan, 1996; Winking and Koster, 2015). Again, this raises the possibility that signaling resource availability (a direct benefit) through conspicuous consumption is what the women in the studies by Sundie et al. (2011) found attractive, even in potential short-term partners, and that men were compelled to signal prestige as an indicator of wealth to attract mates. Unsurprisingly, it has been noted that selection for indicators of prestige closely resembles sexual selection (Richerson and Boyd, 2008).

Cross-cultural studies on mating preferences also support the hypothesis that human mating choices are guided by direct phenotypic benefits, meaning that people generally choose potential partners on the basis of immediate returns, such as resource allocation, parental investment, disease avoidance, status, fertility, etc. Men, for instance, have been observed to show preference for young women with a low waist-to-hip ratio – which are cues of imminent fertility and good general health (Zaadstra et al., 1993; Buss, 1994; Singh, 2002). Women, for their part, tend to prefer men who are expected to provide resources, protection, and/or parental care (Geary et al., 2004; Todd et al., 2007). This means that the correlation between certain behavioral traits and mate choice may still hold, but on the grounds of direct returns, not genetic fitness. The types of direct benefits that are preferred, though, should vary across cultural contexts.

Furthermore, we should consider that in many societies throughout history, mate choice has not always been a matter of free will and individual decision-making based on personal preferences. On the contrary, it is quite likely that the social mediation of reproduction and the institutionalization of sexual relations happened early in human evolution (Harris and Ross, 1987; Knight, 1995; Deacon, 1997; Dunbar and Shultz, 2007), which would imply that mate choice has been long bound to cultural normativity (restrictions on marriage, exogamy/endogamy patterns, offspring affiliation and kinship rules). In kin-based societies, human action is generally compelled to follow social roles and expectations, hence we must specially consider the influence of the social system on mating behavior instead of the reverse (Meillassoux, 1972). A great deal of anthropological data highlights the relevance of the social environment in human sexual selection, particularly the influence of parental and close kin preferences (Apostolou, 2007; Buunk et al., 2010). In arranged marriages, for example, where kin or parental choice is predominantly exercised, traits related to good genes such as physical attractiveness are generally less important, while traits related to direct returns, such as being a hard-worker and coming from a good family, are highly preferred (Apostolou, 2007).

A final problem with the Zahavian model of art is that it assumes that over human evolution mate choice followed the pattern of male display and female choice, which is characteristic of polygynous species (like the bowerbird, and lekking birds like the peacock). In contrast, the evidence suggests that in fact female hominins have been more prone to selective pressures for the physical and energetic requirements of bearing increasingly larger, big-brained babies. This is particularly evident in the marked escalation of female size from *Australopithecus* to *Homo erectus* and onwards (Aiello and Key, 2002). In turn, this suggests that natural selection pressures in response to changing environments and nutritional stress were probably more significant than mate choice preferences in shaping the reproductive anatomy and behavior of the two sexes in our genus (Pawlowski, 1999).

In general, our understanding of human mating systems indicates that human reproductive strategies are widely varied and flexible, changing according to specific cultural, ecological, and economic circumstances (Kaplan, 1996; Fuentes, 1999; Wood and Eagly, 2002). Thus, there is little ground to support a single mating pattern as evolutionary prevalent. In fact, the diversity and flexibility of human mating strategies may indicate that sexual selective pressures were not that significant in hominin evolution (Pawlowski, 1999). But even if it is not clear whether mate choice was exercised by males towards females, females towards males, individually or through kin, it does seem that human mate selection consistently favors direct benefits.

## The Handicap Principle: A Conjurer’s Trick

The Zahavian signal model attributes the origin and function of visual art to human mate choice strategies and defines it as a fitness display. Its main hypotheses have been elaborated by looking at some of visual art’s current effects and, to an extent, offer an accurate description of art’s role in human life. There is no denying that visual art practices, from bodily embellishment to artistry, can and do affect human mate choice as noticed by Darwin and his followers (Hirn, 1900). On the other hand, I have suggested that cultural data does not support mate choice for good genes. In this section, I show that this apparent contradiction stems from the origin of the Handicap Principle and argue that even if art does play a role in human mate choice and may in fact function as a costly signal, it is not an indicator of good genes as has often been suggested.

The Handicap Principle, or the model of costly signals as fitness indicators, was first put forward by the ethologist Amotz Zahavi in 1975 and rose to prominence in the following decades, spearheading a revival of sexual selection research (Penn and Számadó, 2020). It was not long before the idea of costly good-genes indicators was applied to human cultural traits, and the arts in particular (Zahavi and Zahavi, 1997).

As previously mentioned, the arts seem to comply with many of the characteristics usually associated with the extreme animal traits that are related to courtship displays, such as antlers and colorful feathers. Often, these are equally described as exaggerated, conspicuous, wasteful, aesthetic, and lacking any apparent survival value. Under such a description, the parallels between animal ornaments and the visual arts seem strong. Consequently, an explanation that would apply to the former may be easily transferred to the latter. The Handicap Principle provides one such explanation. Costly signal or handicap models suggest that when a preferred trait, like an ornament, is energetically costly, only optimal individuals in better condition will be able to afford and maintain a more elaborate version of it. Due to its dependency on the individual’s condition, the ornament will become a reliable indicator of genetic quality (Jones and Ratterman, 2009). Applied to the arts, the rationale is that the time and effort that people invest in artistic practices had better be allocated to subsistence or reproductive activities, unless the arts were understood as costly signals correlated to genetic fitness (Zahavi and Zahavi, 1997).

The Handicap Principle is largely based on the model of conspicuous consumption, or “theory of waste,” formulated at the dawn of the 20th century by the economist Thorstein Veblen in *The Theory of the Leisure Class* (Veblen and Banta, 2007). Veblen observed that in modern society the upper classes will put excessive resources towards goods or practices (including sports and the arts) which serve as social displays of high-status. The elevated costs of these pursuits and their function as markers of prestige become mutually reinforcing, sustaining a wasteful economy based on high status-seeking. In Zahavi’s biological version, the signals are tied to and advertise the individual’s genetic, instead of their economic condition.

As mentioned previously, Zahavi’s theory was primarily put forward as an explanation for the evolution of conspicuous animal traits and behaviors which, Darwin noted, play a key role in sexual selection. Later, it was reapplied from biology to human cultural traits, like the arts. In this way, the origin of costly signaling theory is often mistakenly situated in biology (e.g., Quinn, 2015), whereas it can be traced back to Veblen’s economic theory. By connecting costly cultural signals to genetic fitness and mate choice for indirect benefits in humans, even when evidence is scant, the Handicap Principle has generated much misunderstanding around human mate choice and costly signaling theory (Penn and Számadó, 2020).

The “biologization” of social theories is not uncommon. In Darwin’s time, the philosopher Friedrich Engels already protested the application of Darwinian theory to human affairs, since Darwin had used Malthus’s social theory of population and applied it to nature, only to have it reapplied to society. He complained: “When this conjurer’s trick has been performed, the same theories are transferred back again from organic nature into history and it is now claimed that their validity as eternal laws of human society has been proved” (Marx and Engels, 1950, p. 368). This is precisely the case with the Handicap Principle.

To be clear, this criticism is not directed at either the sexual selection, mate choice, or costly signaling theories. As mentioned above, these have sound foundations and have provided successful theoretical frameworks to explain a wide array of natural and social phenomena. In fact, I have argued elsewhere that understanding art as a signal provides the best definition and framework to explain the evolution and functional versatility of visual art (Straffon, 2016). It is also not a critique to applying biological or evolutionary approaches to human culture or the arts. The present criticism is directed towards the application of the Handicap Principle to human cultural behaviors and specifically to (visual) art because, as I have discussed so far, there is little evidence to support it. On the one hand, human mate choice does not appear guided by seeking long-term indirect benefits, and there is no proof that the visual arts correlate with genetic quality, on the other.

How then, could we explain the “wasteful” investment in artistic activity and its attested influence on human mate choice? I argue that visual art and other cultural traits may well be costly signals, but not related to good genes. That is, they do not act as genetic fitness indicators but rather as social tools to obtain and convey prestige, that is, as conspicuous signals in Veblen’s original sense. As a Veblenian signal, visual art would still have many of the effects suggested for visual art as a Zahavian signal (e.g., attract mates, impress rivals), except not for the indirect benefits of optimal offspring but for the direct benefits of acquiring and conveying a good image and social status. In this manner, visual art might nevertheless play an important role in human mate choice. Either by creating, appreciating, or displaying artworks, people can signal important social cues such as skill, industriousness, education, taste, and economic success (Dutton, 2009). It follows that if visual art conveys social status, and social status is a predictor of human mate choice, then individuals (male and female) will invest in undertaking and judging visual art practices, to influence potential partners.

## Art as a Functional Veblenian Signal

The Handicap Principle has been appealing as an explanation for human art behavior, I believe, for two reasons. First, it provides an actual and accurate definition of visual art as a signal, which can be corroborated by empirical research. Second, it seems to offer an answer to the enduring question of why people devote energy and resources to an activity devoid of any utilitarian purpose (Morriss-Kay, 2010). Below I argue in favor of defining art as a signal but suggest that contrary to the assumption that art is a non-functional behavior, it plays an important role in human interactions.

In previous work I have argued that visual art indeed seems to be best described as a signal (Straffon, 2016). In biological communication, signals are defined as any stimulus (act or structure) that conveys information to organisms and affects their behavior (Otte, 1974). Animal signals are emitted and inform others about, for instance, the identity, presence, state, or intention of the sender, or about an element in the environment (Endler, 1993). Effective signals must be within the hearing or visual range of conspecifics and must be distinguishable against the background to avoid interference, therefore signals are usually under selection to comply with certain properties that increase their detectability, discriminability, and memorability (Guilford and Dawkins, 1991). Some attention-grabbing, memorable components include typical signal properties like redundancy, conspicuousness, stereotypy, contrast, pattern, novelty and exaggeration, which incidentally are often listed as properties of art (Dissanayake, 2007; Dutton, 2009).

Ethologists coined the term “ritualization” to describe the process by which an ordinary movement, gesture, or vocalization becomes a communication signal (Lorenz, 1966). Julian Huxley suggested that over human evolution, the arts had also undergone ritualization, and for this reason they had a lot in common with animal signals such as courtship or aggressive displays (Huxley, 1966). Common elements between artistic creation and ritualization include the operations of formalization, repetition, exaggeration, elaboration, and manipulation of expectation (Erikson, 1966; Dissanayake, 2007). Thus, in the eyes of ethologists, the arts (dance, song, music-making, oratory, poetry, drama, and visual representation) count as a set of human ritualized behaviors, that is, as human signals (Huxley, 1966).

Because signals must stimulate the receiver’s perception, they tend to incorporate and exploit pre-existing sensorial biases and preferences (Verpooten and Nelissen, 2010; Prum, 2012). Consistent with this, visual art grabs and manipulates the viewer’s attention by exploiting and altering the formal properties of materials and objects, such as color, size, texture, and shape, often creating stimuli that display redundancy, rhythm, and exaggeration, which are effectively attended and recalled (Rossiter, 1982; Krebs and Dawkins, 1984). Furthermore, visual art also makes use of cultural systems of affective and aesthetic values to grab attention and increase memorability (Grammer et al., 2003; Levine and Edelstein, 2009; Verpooten and Nelissen, 2010).

Visual art is a successful signal precisely due to the (positive or intense) aesthetic, affective and cognitive responses it induces in the perceiver. It plays with the formal properties of objects to stimulate bio-cultural perceptual biases to make them increasingly detectable, discernible, memorable, and thus effective as signals (Eibl-Eibesfeldt, 1988). In sum, visual art certainly complies with all the characteristics of a communication signal, which means it can be studied through signaling theory. Since the Handicap Principle deals with signals, it seems to apply to the arts particularly well. What I call into question is its attribution of art as a signal of genetic fitness.

Let us now look at the underlying assumption that art-making is not easily explained by natural selection. The Handicap model argues that because natural selection is an economizing process, it would not have promoted the persistence of a costly and apparently unnecessary behavior such as art. Sexual selection, on the other hand, often results in the development of seemingly useless but attractive traits. So, mate choice for indicators of good genes seems to provide an answer to the question of why art evolved and was retained even when it has no practical purpose. The whole argument hinges on the presupposition that art has no function and is impossible to explain through natural selection, leaving sexual selection as the only other possible account.

However, defining (visual) art as a signal automatically refutes the premise that it has no purpose or adaptive value and opens the possibility of understanding its evolution and actual function through “regular” natural selection mechanisms and communication theory. For example, research in non-human animal communication has shown that because sensory systems and signals coevolve, many of the general properties of the signaling systems of a species should be predictable from its environment, general behavior, and neurobiology (Endler, 1993; Johnstone, 2009). Given that visual communication is central to primates in general and to humans in particular (Dunbar, 1998; Tomasello, 2008), and that we are a tool-making species, signaling through artifacts (Wobst, 1977) would be a predictable human behavior. We therefore should be able to study the natural selection pressures that gave rise to artistic behavior in humans, its function, and potential adaptive value.

Most of the behaviors that typify the so-called human evolutionary niche (Whiten and Erdal, 2012) increase fitness by either improving subsistence and resource acquisition, or by increasing survivorship and lowering overall mortality (Kaplan et al., 2000; McBrearty and Brooks, 2000). Thus, if visual art somehow contributed to fitness throughout human evolution, its adaptive effects should be found in these spheres, rather than in mate choice. If visual art is, as I have suggested, a Veblenian social signal of status, we should then ask why signaling status matters for humans. Elsewhere, I have argued that visual art may have originated in personal ornamentation to advertise identity across social networks (Straffon, 2016). By advertising identity, visual art could have enhanced the fitness of the humans who engaged in it by facilitating cooperation with allies (thereby improving resource acquisition), and by lowering the risk of conflict with strangers (thereby decreasing mortality risks). This provides an ultimate explanation for the evolution of art. As an effective social signal of group membership and status, people should be willing to engage in making and consuming visual art despite its costs. This offers a proximate cause.

Conceiving of visual art as a Veblenian social signal offers both a definition and a framework to understand its evolution, functions, and effects in human cognition and behavior. In addition, it demystifies art’s origins and allows us to understand visual art as a purposeful practice with potential adaptive value. Finally, it accounts for some ultimate and proximate causes of art, something that the Zahavian explanation has so far failed to do (Penn and Számadó, 2020).

## Art and Evolution: Not by Genes Alone

This account of art as a Veblenian signal is no less evolutionary than the Zahavian model. Moreover, it still relates art to sexual selection through mate choice and predicts the same effects of attracting mates and impressing rivals, except not for the indirect benefits of optimal offspring (good genes) but for direct phenotypic benefits (like prestige, industriousness, or resources), which have been shown to actually affect fitness. As discussed above, mate choice for direct benefits is one of several mechanisms of sexual selection (Andersson and Simmons, 2006), and is more in line with empirical evidence on human reproductive strategies than good-genes models.

If art still affects mate choice, does it matter if it is through genes or culture? It does if we aim at reconstructing the evolutionary history, and hence the ultimate causes, of a behavior (Tinbergen, 1963). Focusing on good-genes models has led researchers to concentrate almost exclusively on the selective pressures of male behaviors and female preferences, leaving some important facts about visual art unexplained. For example, the early onset of artistic skill in ontogeny, the fact that women are just as efficient and prolific in art-making (even if not as publicly successful), the fact that modern artistic success is determined by institutions that are largely constituted by men (the art world, i.e., art critics, collectors, dealers), or that the earliest visual art was likely not directed at sexual mates but at kin and social partners (Straffon, 2016). All this suggests that influencing mate choice is just one of many roles that visual art plays in human life, and that the function of visual art as a costly Veblenian signal encompasses but exceeds mate choice, as evidenced by its relevance in intra-sexual, intergenerational, and kin and outgroup relations.

Explaining art as a costly signal in Veblen’s sense allows us to look for selective contexts of visual art other than mate choice, such as communication, cooperation, and ritual. Moreover, it does not discard the effects of biological evolution. Cultural behaviors can also evolve and be selected as adaptations to the environment (Richerson and Boyd, 2008), and can shape genetic information by influencing which genes are favored by natural selection (Heyes, 2018).

## Conclusion

Throughout this paper, I have argued that applying Zahavi’s Handicap Principle to explain the evolution and function of the arts, and particularly of visual art, is a flawed endeavor. I have given three main reasons. First, I have suggested that the underlying argument that humans exercise mate choice for indirect benefits (or good genes) lacks support from empirical research, historical, and cross-cultural data.

Second, I have discussed that the Handicap Principle is a biologized version of Veblen’s “theory of waste,” which already provided an explanatory mechanism for human conspicuous signaling. Thus, we may keep the conceptualization of artistic practice as a costly signal, without having to invoke any association to good genes. Instead, we may understand art as social tool to obtain and convey prestige, that is, a conspicuous signal in Veblen’s sense. As a Veblenian signal, art may still attract mates and impress rivals, advertising direct benefits such as social status and resources and, because these impact fitness they are important factors in mate choice, so individuals (male and female) should want to invest in visual art in order to influence potential partners. In this manner, art would still play an important role in human mating.

Finally, I have proposed that the Handicap Principle has been easily applied to the arts because it defines art as a signal, which is a fitting description. However, by keeping to a misguided definition of art as a non-purposive behavior, the Zahavian model excludes the possibility of approaching it through natural selection. Whereas, like any other animal signal, art likely evolved under natural selection pressures, and has played an important function in human communication since its origin.

## Author Contributions

The author confirms being the sole contributor of this work and has approved it for publication.

## Funding

This work was partly supported by the Research Council of Norway through its Centres of Excellence funding scheme, SFF Centre for Early Sapiens Behaviour (SapienCE) project number 262618, and by the John Templeton Foundation (grant number 61403).

## Conflict of Interest

The author declares that the research was conducted in the absence of any commercial or financial relationships that could be construed as a potential conflict of interest.

## Publisher’s Note

All claims expressed in this article are solely those of the authors and do not necessarily represent those of their affiliated organizations, or those of the publisher, the editors and the reviewers. Any product that may be evaluated in this article, or claim that may be made by its manufacturer, is not guaranteed or endorsed by the publisher.

## Footnotes

^1^Sexual selection is not limited to mating competition, but that is what Darwin focused on when he came up with the term and it remains its most simple and useful application (Andersson, 1994).

^2^Alternatively, the peacock’s tail may be an armament for intrasexual competition, shaped by male-male conflict instead of female choice (Berglund et al., 1996), or it could be an ancestral trait that has been lost in the females (Takahashi et al., 2007).

^3^For a history, detailed description, and assessment of the Handicap Principle, see Penn and Számadó (2020).

^4^For example, the preference of female guppies (fish) for more intense orange-tailed males might be traced back to a wide-species feeding preference for orange fruit (Rodd et al., 2002).
